# Supplementary figures and images for: A spatially variant high-order variational model for Rician noise removal
Source: PeerJ Comput Sci. 2023 Sep 26;9:e1579. doi: 10.7717/peerj-cs.1579 (PMC10557481; doi:10.7717/peerj-cs.1579)

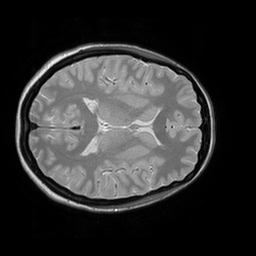

Supplement: Supplemental Information 1 — Image source credit: IXI dataset, CC BY-SA 3.0 (https://brain-development.org/ixi-dataset/). [file peerj-cs-09-1579-s001.zip › SVHOVM/test_image.png]
